# The promise of cerebrospinal fluid biomarkers in idiopathic normal pressure hydrocephalus

**DOI:** 10.3325/cmj.2024.65.303

**Published:** 2024-08

**Authors:** Romana Perković, Fran Borovečki

**Affiliations:** Division for Neurodegenerative Diseases and Neurogenomics, Department of Neurology, University Hospital Center Zagreb, Zagreb, Croatia

With population aging and a surging prevalence of neurodegenerative diseases, clinical neuroscience requires new, low-risk methods for diagnostic and prognostic purposes. Idiopathic normal pressure hydrocephalus (iNPH) is increasingly encountered in everyday clinical practice. As in these patients a delayed diagnosis leads to a worse prognosis, physicians are in constant search for the best diagnostic methods. The standard method of treatment (1) for iNPH is cerebrospinal fluid (CSF) diversion through a surgically placed shunt, and one of the best known prognostic approaches for shunt efficiency is external lumbar drainage (ELD).

However, even if all the procedures are strictly followed, each patient will require an individual approach. A good result may be absent after removing ELD, but the patient may still respond well to the shunt, and *vice versa*. In many cases, the usefulness of shunt placement is evaluated empirically without additional objective parameters. To further standardize the process and eliminate surprises, ELD should be combined with non-invasive procedures such as assessing biomarkers from CSF. The neuropathology of iNPH includes severe degeneration of periventricular areas, and neurodegenerative processes inevitably lead to changes in CSF composition (2).

There is a need for biomarkers that could distinguish iNPH from other clinical mimics, especially Alzheimer’s disease (AD). It is well established that the concentration of amyloid-β (Aβ1-42) in CSF is notably lower, and the concentration of total tau (tau) and phospho tau (p-tau) is higher, in AD patients than in healthy controls. Various studies have investigated biomarkers for iNPH, but none of the biomarkers are currently in clinical use. Also, 25%-40% of iNPH patients exhibit AD (1).

The study by Brgić Mandić et al (3) published in the current issue of the *Croatian Medical Journal* hypothesized that changes in CSF concentrations of Aβ1-42, tau, and p-tau may help in the prognosis of response to treatment in patients with iNPH. The concentration of all the tested biomarkers increased during the first 36 hours of ELD. Aβ1-42 levels were higher in responders than in non-responders at all measurement points, with a significant difference after 72 hours. Respondents also had a significantly higher Aβ1-42/Aβ1-40 ratio at all time-points.

The findings by Brgić Mandić et al are extremely interesting since some of the previous studies showed that shunt-responders had lower lumbar CSF concentrations of tau and p-tau proteins than non-responders, with no significant difference in Aβ 1-42 levels (4). Some studies showed decreased levels of Aβ 1-42 (2), while others observed increased levels of Aβ 1-42 and tau proteins (5). These apparent discrepancies may be attributed to variations in analytical methods, methodological weaknesses, a small number of studies dealing with Aβ 1-42 changes, and different CSF sample collection times after varying drainage durations. Such data should, therefore, be interpreted cautiously with sufficient comparative variables, especially taking into account that CSF biomarker levels in patients with iNPH may be affected by an inherent reduction in brain volume (5). Finally, the best prognostic value might be obtained by using a combination of several CSF biomarkers (6).

In addition to predicting the benefit of shunt placement after ELD, biomarkers could be used to monitor the progression of the accompanying cognitive decline and determine the long-term prognosis of patients with iNPH. Ultimately, an optimal biomarker should be able to tell us whether the patient has reached the tipping point in the course of the disease when there will no longer be any benefit from shunt placement.

Significant preclinical progress has been made in establishing the prognostic factors for iNPH, but to transfer these findings into everyday clinical practice, studies on larger cohorts are needed. Over the coming years, progress is expected in the treatment of neurodegenerative disorders, and one of the main requirements for successful application of novel therapies will be the development of suitable biomarkers. The work by Brgić Mandić et al elegantly demonstrates that using the known biomarkers for neurodegenerative diseases in a novel setting may give us just the results we require (3).
